# Patient Perspectives of Barriers and Facilitators for the Uptake of Pharmacogenomic Testing in Veterans Affairs’ Pharmacogenomic Testing for the Veterans (PHASER) Program

**DOI:** 10.3390/jpm13091367

**Published:** 2023-09-09

**Authors:** Karina Melendez, Diana Gutierrez-Meza, Kara L. Gavin, Esra Alagoz, Nina Sperber, Rebekah Ryanne Wu, Abigail Silva, Bhabna Pati, Deepak Voora, Allison Hung, Megan C. Roberts, Corrine I. Voils

**Affiliations:** 1 Department of Surgery, University of Wisconsin School of Medicine and Public Health, Madison, WI 53792, USA; karinamelendez414@gmail.com (K.M.); gutierrez-meza@surgery.wisc.edu (D.G.-M.); ealagoz@wisc.edu (E.A.); bpati@wisc.edu (B.P.); hung@surgery.wisc.edu (A.H.); 2 Center of Innovation to Accelerate Discovery and Practice Transformation, Durham Veterans Affairs Medical Center, Durham, NC 27705, USA; 3 Duke Department of Population Health Sciences, Duke University School of Medicine, Durham, NC 27701, USA; 4 VA National Pharmacogenomics Program, Department of Veteran’s Affairs, Durham, NC 27705, USA; ryanne.wu@duke.edu (R.R.W.);; 5 Department of Medicine, Duke Precision Medicine Program, Duke University School of Medicine, Durham, NC 27599, USA; 6 Center of Innovation for Complex Chronic Healthcare, Edward Hines Jr. Veterans Affairs Hospital, Hines, IL 60141, USA; 7 Parkinson School of Health Sciences and Public Health, Loyola University Chicago, Maywood, IL 60153, USA; 8 Division of Pharmaceutical Outcomes & Policy, Eshelman School of Pharmacy, University of North Carolina at Chapel Hill, Chapel Hill, NC 27599, USA; megan.roberts@unc.edu; 9 William S. Middleton Memorial Veterans Hospital, Madison, WI 53705, USA

**Keywords:** veterans, PGx testing, pharmacotherapy, enablers, facilitators, barriers

## Abstract

We applied implementation science frameworks to identify barriers and facilitators to veterans’ acceptance of pharmacogenomic testing (PGx), which was made available as a part of clinical care at 25 VA medical centers. We conducted 30 min interviews with veterans who accepted (*n* = 14), declined (*n* = 9), or were contemplating (*n* = 8) PGx testing. Six team members coded one transcript from each participant group to develop the codebook and finalize definitions. Three team members coded the remaining 28 transcripts and met regularly with the larger team to reach a consensus. The coders generated a matrix of implementation constructs by testing status to identify the similarities and differences between accepters, decliners, and contemplators. All groups understood the PGx testing procedures and possible benefits. In the decision-making, accepters prioritized the potential health benefits of PGx testing, such as reducing side effects or the number of medications. In contrast, decliners prioritized the possibilities of data breach or the negative impact on healthcare insurance or Veterans Affairs benefits. Contemplators desired to speak to a provider to learn more before making a decision. Efforts to improve the clarity of data security and the impact on benefits may improve veterans’ abilities to make more informed decisions about whether to undergo PGx testing.

## 1. Introduction

Pharmacogenomic (PGx) testing aims to improve health care delivery of medications by increasing efficacy and reducing adverse drug events through altering a dose or type of medication to align with a patient’s genetic test results. Current evidence supports PGx testing to improve therapies for cancer, mental health, pain, cardiovascular, and inflammatory disorders [1]. A study of over 73 million patients in the United States suggested that one-third could benefit from PGx testing based on the pharmacogenomic guidelines of one or more medications [2].

As PGx testing becomes more widespread, understanding the factors that impact patient uptake is imperative. Some insight may be gained from studying the experiences of patients who have undergone pre-emptive genetic testing. For example, Lemke conducted a survey of primary care patients who received broad pre-emptive genetic testing for hereditary conditions, ancestry, PGx, and common trait information [3]. They found that the majority indicated that the desire to learn about their personal response to medication drove the decision to get tested, whereas only one-third reported that family member recommendations influenced their decision. Three weeks after testing, nearly half of the participants reported concerns about the privacy of their results, and one-third reported concerns about the impact on their health and life insurance. Although these results are informative, barriers to PGx testing may differ from barriers to genetic testing for other reasons, such as hereditary disease or ancestry. 

Fewer studies have been conducted about barriers and facilitators to PGx testing. A previous focus group study comparing patients who were offered and not offered preemptive PGx testing found that both groups believed that PGx testing could inform physicians’ prescribing to improve efficacy and reduce side effects. Both groups also expressed desire for physicians to discuss risks and benefits with patients during decision-making. Both groups also expressed concerns about how testing would impact insurance coverage and employment discrimination, as well as who would be permitted to access the results. Participants who were not offered testing expressed skepticism about how the results would be used [2]. In a survey of largely minority patients followed at an antithrombosis clinic who were prescribed warfarin, facilitators of PGx testing included beliefs that PGx testing would be beneficial for their health, trust in providers, insurance coverage, and test affordability. Barriers included the potential negative impact of testing, concerns about the test process, lack of interest in PGx, and concerns about the risk, privacy, and reliability of PGx testing [4]. A systematic review addressing the enablers of implementation of PGx testing stated that optimism that PGx testing would benefit healthcare facilitated PGx testing, whereas pessimism about the utility of PGx testing was a barrier [5]. This review also identified the cost and DNA collection methods such as saliva-sampling as patient barriers to receiving PGx testing. These studies were all done in private healthcare settings in which the cost of testing is a potential barrier. Less is known about facilitators and barriers to PGx testing when cost is not an issue. 

The Veterans Health Administration (VHA) is the largest integrated healthcare system in the United States with over 9 million veterans enrolled. The VHA began offering preemptive, panel-based PGx testing for 10 genes that inform the prescribing of 40 commonly prescribed medications at no cost to patients through the Pharmacogenomic Testing for Veterans (PHASER) program in 2019 [6]. Based on estimates from the VHA pharmacy data, 1 in 2 veterans will be newly prescribed a medicine that could be informed by the PGx test results over a 6-year period [7]. Furthermore, 99% of VHA pharmacy users are projected to carry at least one actionable PGx variant that leads to medication adjustments [7]. 

Given the need and use of VHA healthcare users, the VHA implemented the PHASER program to create the infrastructure needed to support panel-based PGx testing with the goals of educating patients and providers on the use of PGx in clinical care; creating the data and informatics tools needed to integrate PGx into clinical practice; and reducing the risks of adverse drug events by better aligning prescriptions with the genetic test results. The goal of the program is to offer veterans PGx testing of up to 40 commonly prescribed medications to provide information about drug dosing, toxicity, and effectiveness by the end of 2024. As of June 2023, there had been approximately 30,592 orders. 

Each PHASER site has a physician and pharmacy site champion who interface with the national program. These individuals lead a steering committee of additional local prescribers and pharmacists. The steering committee develops the outreach strategy for their facility, which can range from general education to providers regarding PGx testing and how to order for their patients; health system-wide, direct-to-Veteran educational outreach; the use of pre-appointment educational mailings for patients with upcoming appointments; or direct patient telephone calls. The latter two modalities required that providers “opt-in” to these services for their patients. These interactions are meant to educate patients about the availability of PGx testing, potential benefits and limitations, and to encourage them to request testing from their providers if interested (mailings, direct-to-Veteran) or to consent to testing directly (telephone calls) with an order placed for the patient’s provider to sign. Each site was free to choose from all available options for educating patients and providers about the availability of PGx testing. Each site also receives funding for at least one year for a project coordinator who notifies the ordering provider when the results are available in the electronic medical record and mails as an excerpt of the results to the patients.

The present work is part of a larger program evaluation to identify facilitators and barriers to PGx testing at the patient, prescriber, and healthcare system levels. By understanding multiple perspectives about the uptake of PGx testing, we aim to identify actionable factors that affect participation in PGx testing within the VHA. Herein, we report on theory-driven qualitative interviews with patients who have accepted, have declined, or are still contemplating receiving PGx testing. 

## 2. Materials and Methods

### 2.1. Design, Setting, and Participants 

This study involved one-time interviews with veterans who had been approached about PGx testing between January and October 2022. Veterans were recruited from one of the 25 VA sites participating in the PHASER program during those dates. At these sites, a data field was entered into the electronic medical record indicating whether patients had accepted or declined testing. An “Accepted” indicator was required to be populated in order for a PGx test order to be placed. “Declined” could be populated by providers who offered patients testing or by site coordinators who sent patients a letter about PHASER and called them to discuss participation. Neither coordinators nor providers were required to document declines, however. Using the variable populated as accepted or declined, we purposefully sampled patients, aiming to interview and recruit sufficient numbers of patients in each group to achieve informational redundancy. 

Patients needed to have a valid phone number and email address to be eligible for the interview. We mailed invitations to veterans meeting the eligibility criteria to participate in a one-time, 30 min interview about the PHASER program. At least one week later, we called to schedule them for an interview. We intentionally recruited from various sites and sought variability in gender and race.

During interviews, we learned that some veterans who were categorized as decliners had not actively declined testing. They reported that the recruitment letter they received about the interview and subsequent call from the research team was the first information they had received about PHASER and indicated that they would consider being tested. Therefore, we re-categorized this group as contemplators (i.e., people who learned about the program through the evaluation team and had not yet made a decision about whether to have testing). The interviewer started the contemplator interviews similar to the other interviews by asking about their understanding of PGx testing. The interviewer provided a definition of PGx testing mainly when contemplators asked what testing entails. Since most contemplators had no understanding of PGx testing, the interviewer provided background about PGx: “The VA is offering pharmacogenomics testing to veterans. To get tested, your provider orders a blood test that looks into your genetic information to pick medications that may work best for you”. The interviewer did not provide an in-depth education about PGx testing. If further questions and interest were expressed, the interviewer directed the patient to contact their VA provider and informed the study coordinator about their interest. 

### 2.2. Procedures 

We developed interview guides (Table 1) using the Theoretical Domains Framework (TDF) [8]. The TDF assesses 14 domains that outline implementation processes, including individual-level determinants [8]. We developed the accepter interview guide using a subset of determinants the team felt were important for informing PGx testing based on the previous literature [5] or our content expertise. We pilot-tested the accepter interview guide with three veterans who accepted testing, refining it and using the final version for all subsequent accepter interviews. We then modified the questions for decliners. Questions for contemplators were modified to be hypothetical, asking participants what they understood and would need to know more about in order to decide whether or not to have testing.

### 2.3. Analysis 

Using directed content analysis [9], six team members coded the first transcript from each patient group (i.e., accepter, decliner, and contemplator) to refine the TDF constructs and definitions and add emergent themes that were not captured in the TDF. After the team consensus of the codebook and definitions was reached, the remaining transcripts were coded by one of the three team members with the finalized codebook. The data section coders who could not reach a consensus were brought back to the full team to review and resolve. Queries for each construct were then generated and distributed among the four team members to write summaries describing the themes based on each patient group. Finally, the summaries were combined to compose thematic narratives of how each construct was represented among all three patient groups. The narratives were presented to the qualitative evaluation team at all-group qualitative team meetings to ensure that all aspects of veterans’ decision-making in participating in the PHASER program were captured. These “thick descriptions” of themes, along with having multiple coders and regular peer reviews, ensured our results were grounded in the data and established the reliability of our results [10].

## 3. Results

**Figure 1:**
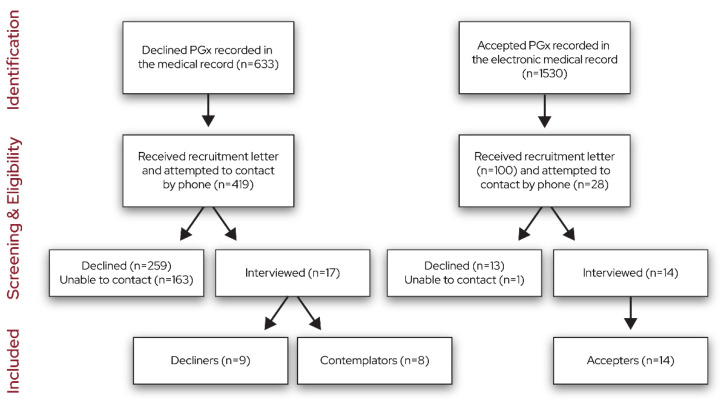
Recruitment flow diagram.

The recruitment flow diagram is shown in Figure 1. We stopped interviewing once the team determined that informational redundancy was achieved in each group via discussions during data collection and the generation of the data summaries. We interviewed 14 accepters, 9 decliners, and 8 contemplators. 

Table 2 shows self-reported demographics. Most participants were male, white, non-Hispanic, and had completed some college. Half of the participants were retired, and half were married. The average age was 60 years. 

Next, we describe the findings via the TDF domain. Illustrative quotes are provided in Table 3. 

### 3.1. Knowledge

Accepters and decliners had knowledge about the rationale for PGx testing, namely, that it could be used to help their provider select medications that would improve efficacy, reduce side effects, and reduce trial and error. Procedural knowledge, a sub-domain of knowledge, referred to how to get the testing and the results. Accepters and decliners had some level of procedural knowledge about what was involved with getting PGx testing. Accepters stated that easy-to-follow instructions on how to get PGx testing allowed them to complete the genetic test during their routine bloodwork. Several decliners and one contemplator anticipated the procedures would be time-consuming, citing time to set up the appointment, to travel to and from the VA hospital, and to complete the PGx test. Some contemplators mentioned confusion about the logistics of receiving the PGx test: They were not sure how to talk to providers about the program and were unaware that the test would require a blood test or where to learn more information about the program. 

Knowledge of the task environment, a sub-domain of knowledge, referred to the understanding of others’ access to the PGx results. With the exception of one accepter who reported that information could be accessed without permission via cyberattacks, accepters generally thought that only licensed professionals would have access to this information and that it would be stored safely. Two accepters saw it as a benefit that the results could be accessed by providers they do not normally see, such as when they are traveling and need to see a provider at another VA hospital. A number of decliners raised concerns about the safekeeping of their genetic information. They were apprehensive of their personal information being disclosed to third parties. One decliner mentioned that their distrust in handing over their genetic information was related to direct-to-consumer businesses selling participants’ information. Some contemplators also mentioned worries about storing their private health information, whereas others believed their information would be kept safe and confidential. 

### 3.2. Memory, Attention, and Decision Processes

Memory, attention, and decision processes dealt with how veterans weighed the benefits and drawbacks in making a decision about whether to get tested. All three groups mentioned potential benefits and drawbacks of PGx testing. Accepters and decliners weighed the benefits and drawbacks differently, influencing their decision to receive or not receive testing. Whereas accepters considered the opinions of others and focused on the potential benefits of testing to their health, the decision-making of decliners was driven by perceived negative consequences such as the disclosure of information or the negative impacts on their healthcare benefits. Contemplators reported not feeling knowledgeable enough to weigh the benefits and drawbacks to make a decision.

### 3.3. Social Influence

Social influence included perceptions about how providers, family, and friends impacted veterans’ decisions to get PGx testing. Accepters mentioned that clear communication with a provider encouraged them to get PGx testing provided by the PHASER program. They directly asked providers about how the PGx test could benefit their care and what procedures they should follow to receive the test. Accepters reported that providers emphasized veteran autonomy in choosing whether to have PGx testing. In contrast to the positive influence of providers on accepters, several decliners indicated that providers did not influence their decision-making process. A few decliners mentioned that their past education and personal research on the topic allowed them to feel secure in deciding to decline the test. Moreover, one decliner stated that once a provider did bring it up, they did not want to continue the conversation and asked to change the subject. Two contemplators stated that they would like to talk to a provider about the PHASER program in the near future to help them make a decision.

In several cases, family and friends also influenced participant decision-making. Some accepters said that family and friends emphasized the possible benefits of testing for the patient’s health goals, whereas others mentioned that family or friends emphasized that veterans should make their own choice about whether to undergo testing. Two accepters said the opportunity to help other veterans supported their decision to receive the PGx testing. Several decliners and contemplators indicated that family and friends did not influence their decision-making process. 

### 3.4. Optimism

Optimism refers to confidence that things will go well, the healthcare system acts with veterans’ interests in mind, and providers will make the best decision for veterans. Accepters saw PGx as an advantage to help their providers find better solutions to their health problems. In contrast to optimism is pessimism, the belief that things will go wrong, or that the VHA and providers do not act in veterans’ best interest. Only decliners expressed pessimism that negative consequences would arise from PGx testing. 

### 3.5. Environmental Context and Resources

Environmental context and resources includes any aspect of a person’s situation or environment that discourages or encourages the person from receiving PGx testing. Accepters’ discussions about environmental context related to the ease of testing. They noted that testing was a smooth process, like any other lab test. Decliners’ discussion of environmental context was about distrust in the government and the VHA due to historical events such as testing on African American individuals and military personnel. One decliner opined that authoritative figures within the justice system could use this information against them or to harm the veteran and their family. This same person thought that future technology could lead to the misuse of their genetic data in a negative way. One decliner felt there was a risk of negatively impacting their private healthcare and long-term insurance. 

### 3.6. Beliefs about Consequences

Beliefs about consequences refers to the costs and consequences to the veteran for getting PGx testing. Accepters, decliners, and contemplators were largely aligned in their belief that PGx testing could reduce trial and error to find the right medication. Accepters and decliners hoped that PGx testing could result in them taking fewer medications. Accepters believed that testing could result in fewer side effects, an optimal dose, and living better with their health problems. One accepter noted as a drawback that the testing would not specify which medication should be used. One veteran declined PGX testing because she believed it would not benefit her because she has no children. Another decliner stated that PGx testing would not be informative because they were too old to benefit. Another veteran declined because PGx would only help themselves, not other veterans. Other decliners explained that they did not see the benefits extending beyond their current healthcare goals to their long-term goals. Two contemplators said they were unsure about how PGx testing could benefit them.

### 3.7. Emotion

Participants were asked about how emotion related to their experience or the possibility of PGx testing. Several accepters reported feeling grateful that the VA was funding the program for the health of veterans and would be willing to be tested again, if needed. A few accepters mentioned that they felt dissatisfied with the results of their PGx test because it required a provider to translate the medical jargon. Decliners mentioned anxiety related to the possible disclosure of information.

### 3.8. Outcomes of Testing

All accepters recollected that they had been tested. A few accepters were not certain if anything came of the testing because the providers did not explicitly mention changes to the medications or explain that their current medication and dosages are consistent with the test results. One accepter felt that the testing was not beneficial because it did not lead to any changes in medications. Another accepter felt that the results were not specific enough about which medication would be best for them. A different accepter reported that the PGx results led to an adjustment in medications and felt reassured about her past experience with pain management medications. 

### 3.9. Intentions 

Intentions refers to a conscious decision to have PGx in the future, either again or for the first time. Two accepters who did not know how the testing had affected their healthcare reported that they would not receive testing again. Several decliners stated that they do not intend to receive the PGx test until there is transparency on how the VHA is storing and securing their genetic information. Two decliners reported they do not intend to get testing because they do not believe it will benefit their care. One contemplator, after learning about PGx testing from the interviewer, stated that they do not know how the test would benefit their healthcare since they are not currently prescribed medications. Two other contemplators asked the interviewer to connect them to someone so that they could get testing.

### 3.10. Suggestions for PHASER Program Improvement

All participants were asked to provide suggestions for improving the PHASER program. The suggestions centered around improving education for veterans about testing procedures, possible outcomes, the safekeeping of data, and access. One accepter requested more education aimed at medical providers as well as information on the progress of the PHASER program. A few accepters mentioned that the test needs to be more accessible. One accepter reported that the test should be more inclusive of other medicines. Two decliners noted that the VA should provide more information about PGx testing via different formats such as in-person discussions and mailed letters. They suggested discussions should be more personal rather than clinical. Two contemplators mentioned that the VA should make sure patients receive appropriate information and follow-up to ensure they understand. Some contemplators preferred discussion with a provider to learn about the pros and cons of PGx testing. Others preferred a letter, as long as it explains PGx testing well. 

## 4. Discussion

We found that one’s knowledge of the benefits and risks of the PGx test, previous experiences with the VA, perceptions about the history of research on military personnel, social influences, and communications contributed to veterans’ decisions about whether or not to participate in the VHA’s PHASER program. Facilitators undergoing testing included beliefs that PGx testing would improve medication response and reduce the number of medications. The decision to undergo testing was also facilitated by providers, family members, and friends who reinforced veteran autonomy. Barriers to testing included beliefs that testing would not be beneficial, the time required to get testing, and the possible negative effect on healthcare insurance coverage. These findings are consistent with previous studies [2,4,5]. Also consistent with previous research, we found that veterans who were tested reported largely positive emotions [3]. We also found unique barriers owing to the setting in which we conducted this study, including unclear procedures on the safekeeping of veterans’ genetic information, a history of unjust research on military and minority populations, and varying resources and communications from providers about the PHASER program. Previous research has also identified a history of unjust research and privacy concerns as barriers to genetic testing. These concerns among civilian populations may be compounded with the unique aspects of military service (e.g., compulsory vaccines) and the determination of service connection for the VHA healthcare benefits. 

When attempting to recruit participants who declined the testing, the team identified that there was no clear documentation in the electronic medical record of whether a veteran declined testing. In turn, the contemplator group emerged, providing important information in a group of veterans that could be targeted for program outreach. Individuals still contemplating whether to receive testing reinforced the need for outreach by providers rather than program mailings so that veterans could have their questions answered by a trusted individual. These individuals required more information about the benefits, drawbacks, and logistics to make a decision about whether to receive testing. Contemplators identified that there should be more accessible information and communications from providers about the PHASER program.

This study has several limitations. This evaluation identified the experience of a select few veterans. Qualitative research is intended to sample for possibility; in this case, identifying factors important for decision-making about whether to undergo PGx testing [11]. In some cases, an idea was generated by only one participant, raising the question of prevalence of the facilitator or barrier. These findings can be used to develop a survey to assess the prevalence of these factors in a large, representative sample. Although our sample size was small relative to a quantitative survey study, we achieved informational redundancy, and our sample size was in line with the recommendations for qualitative research [12,13]. Lastly, many of the participants interviewed identified as non-Hispanic, white, retired, and male, which is consistent with the demographics of the larger VHA population. Different perspectives may have been gained from other individuals. This work addresses the perceptions of veterans about PGx testing via the VHA. Further investigation is needed on the perspectives of prescribers, who veterans identified as influential in their decisions about whether to undergo testing. 

These results suggest several targets for modifying the experience for veterans. First, the consent process should address any impact that testing could have on benefits inside and outside the VA. Although the Genetic Information Nondiscrimination Act of 2008 was designed to offer some protection from discrimination due to genetic information, it does not protect individuals from genetic testing impacting their long-term health insurance, nor does it cover veterans who are receiving care from the VHA and any benefits conferred to them through the Department of Veterans Affairs. Relatedly, veterans should be reassured, to the extent possible, about who can access their information and about the steps the VHA takes to ensure data security. These findings highlight the need to find other ways to offer PGx testing, given that some decliners cited time for testing as a barrier. Potential solutions include mobile phlebotomy or saliva-based testing to reach rural veterans and those with other barriers to traveling to a clinic to obtain testing. Finally, veterans should receive clear education about what PGx testing can and cannot do. This will require the education of prescribers who are explaining testing to patients and ordering the test. 

## 5. Conclusions

These results suggest that accepters and decliners are aware of the benefits, risk, and logistics of PGx testing provided by the PHASER program. These two groups weighed the benefits and risks differently. Whereas accepters emphasized the potential benefits to their health goals, decliners emphasized the possibility of data breaches, the negative impact of genetic information on insurance coverage, and the misuse of private health information as influential to their decision on not receiving the test. An unexpected group, contemplators, emerged from these interviews and emphasized the need for provider outreach. In conclusion, participant facing information and recruitment processes should emphasize how PGx testing can benefit veterans’ care and the procedures of safekeeping participants’ private health information.

## Figures and Tables

**Table 1:** Theoretical Domain Framework (TDF) domain, subdomain, definition in this context, and interview question by participant group.

Domain	Subdomain	Definition	Interview Question	Participant Group
Knowledge	Knowledge	Level of understanding of genetic testing and how it works. Discussion of how patient learned about genetic testing.	What do you know about using genetic information to pick a medicine?	Accepter, decliner, contemplator
			What are your thoughts about using genetic testing to pick your medicines? What concerns do you have?	Contemplator
			What would you like to know about genetic testing for medicines?	Contemplator
	Procedural knowledge	Knowing how to get testing done and how to get results.	How did you learn about the opportunity at the VA to use genetic testing to choose your medicine? Tell me more about your discussion with your healthcare team.	Accepter, decliner
			How would you like to learn about genetic testing for medicines to make an informed decision? Do you have a preference for what method, for example, letter, in person, phone call?	Contemplator
	Knowledge of task environment	Understanding of who will have access to their testing results (how it will be stored, how it will be accessed, for how long).	What is your understanding of who will have access to your information? What about how they will access it? Tell me about any reservations you have about safekeeping of your information. Probes: What concerns do you have about the privacy of your results? What concerns do you have about the effect on your benefits or insurance coverage?	Accepter, decliner, contemplator
			What is your understanding of who would have access to your results if you were to get PGx testing? Do you have any concerns about how PGx results will be kept?	Contemplator
Goals	Goals (distal/proximal)	Mental representations of outcomes or end states that an individual wants to achieve	How does testing fit your personal health goals, if at all?	Accepter
			How does your decision relate to your personal health goals, if at all?	Decliner
			(After brief description of PGx testing) What do you see as the benefits to your health?	Contemplator
Memory, attention, and decision processes	Decision-making	Process for choosing between two or more alternatives. How did patients make the decision to get tested or not? Or how would they make the decision?	How did you decide to get the test?	Accepter
			How did you decide not to get the test? (Probe about logistics such as travel time, transportation, time off work, informational resources)	Decliner
			What would make you consider getting PGx testing? Are there other factors that would influence your decision to get testing or not?	Contemplator
Social influences	Social norms	Who or what influenced the decision to get tested	Who or what influenced your decision to get testing/not get testing? (Probes: conversations with the healthcare provider, friends and family, people in your community, media or printed information)	Accepter
Optimism	Optimism	The confidence that things will happen for the best and that the VHA and providers act in veterans’ best interest.	How likely do you think it is that genetic testing will lead to better decisions about finding the right medicine for you? How likely do you think it is that genetic testing will lead to better decisions about improving your overall health?	Accepter, decliner
	Pessimism	Attitude that things will go wrong and that VHA and providers do not act in veterans’ best interest.	Emerged unprompted	Emerged unprompted
Environmental context	Person x environment interactions Salient events/critical incidents Environmental Stressors	Any circumstances of a person’s situation or environment that discourages or encourages the development of skills and abilities, independence, social competence, and adaptive behavior. Environment Can include access to VA clinics, family, justice system, testing on veterans/racial–ethnic minority groups.	Tell me about anything that made it easy for you to get the test?	Accepter
			Tell me about anything that made it difficult to get the test.	Accepter
Beliefs about Consequences	Outcome expectancies	Discussion of what the consequences (drawbacks or benefits) would be if they got PGx testing.	What do you see as the benefits to you, if any, of using genetic information to make decisions about your medicines? What do you see as drawbacks?	Accepter, decliner
Intentions		A conscious decision to perform a behavior or a resolve to act in a certain way. Intent to be tested again or for the first time in the future.	Knowing what you do now, would you consider genetic testing for other medicines in the future?	Accepter
			Knowing what you do now, would you ever consider genetic testing in the future? If no: What would make you change your mind? If yes: Under what conditions would you consider it?	Decliner, contemplator
Emotions	Positive/ negative affect, anxiety, fear	Fear, anxiety, positive/negative affect as it relates to patient experience with PGx	Now that you have had the testing done, how do you feel about your decision? How does that compare to how you felt before you had the test?	Accepter
			How do you feel about your decision now? How does that compare to how you felt before you declined testing?	Decliner
Outcomes of testing	N/A	Patient perceptions of how testing affected their health care, including prescriptions for medicines	What has happened to your care or medicines, if anything, as a result of having the genetic test?	Accepter
Process improvement	N/A	Suggestions for the VA for how to improve the PGx testing process.	How do you think the VA can improve the genetic testing process or experience for patients?	Accepter
			What can the VA do better to inform other veterans about genetic testing for medicines?	Decliner, contemplator

**Table 2:** Participant Self-Reported Demographics.

Demographic Characteristic	Accepters (n = 14)	Decliners (n = 9)	Contemplators (n = 8)
Gender Male Female			
	71%	89%	88%
	29%	11%	12%
Race White African American Native American/Alaskan Native Asian Native Hawaiian or Pacific Islander	85.7% 7% 7% 7%	55.6% 22% 11.1% 11.1%	75% 25% 0% 0%
	7%	0%	0%
Ethnicity Hispanic or Latino Not-Hispanic or Latino			
	21.4%	0%	12.5%
	78.6%	100%	87.5%
Age (SD)	60.3 (14.3)	64.0 (16.8)	62.4 (20.9)
Education High school graduate or equivalent Some college credit, but no degree Associate’s degree (AA or AS) Bachelor’s degree (BA or BS) Post-graduate work or degree	7% 35.7% 28.6% 14.2% 14.3%	11.1% 44.4% 25% 11.1% 11.1%	12.5% 25% 25% 37.5% 0%
Marital Status Single, never married Married Divorced/separated Widowed	7% 50% 26.7% 14.3%	11.1% 33.3% 33.3% 11.1%	0% 87.5% 12.5% 0%
Household Income $10,000–$19,999 $20,000–$29,999 $30,000–$39,999 $40,000–$49,999 $50,000–$59,999 $60,000–$79,999 $80,000 or more	7.1% 7.1% 21.4% 7.1% 28.6% 7.1% 7.1%	11.1% 11.1% 22.2% 0% 11.1% 11.1% 22.2%	0% 12.5% 25% 12.5% 0% 25% 25%

Note. Two accepters and two decliners declined to answer about their household income.

**Table 3:** Interview quotes from each participant group via Theoretical Domains Framework constructs.

Domain	Subdomain	Accepter Quotes	Contemplator Quotes	Decliner Quotes
Knowledge	Knowledge	Well, what I understood from this test was it basically measures the metabolism of your liver enzymes and how they work with certain medications. But the test also isn’t specific enough to say, I guess, which ones work and don’t. Just maybe how you would process it if it did work, if that makes sense. (nH White female)	I don’t really know much about the genetic testing. I never done it. I know that they prescribe different medications for me, and they try to find the best ones for me, the most effective medicine for me. But as far as the genetic testing, I don’t know much about it. (African American male)	I think this is the program to see what drugs that I would be amicable with based on a genetic, you know, on my genotype as far as how I am receptive to certain drugs to help me for whatever malady that I may have. It is a valid thing, except there are so many things wrong with me that I don’t see what the purpose is for me. (Asian male)
	Procedural knowledge	I have to go in for regular lab work anyway, so they just lumped it in with the rest of my lab work… I mean, it was just another day of going to the VA. (nH White female)	I wasn’t exactly sure what I would have to do. (African American male)	She [primary care provider] gave me a pamphlet with information about the [clinic], and then there was also a page about the PHASER testing. It tells me benefits and improves access to treatment, reduces trial and error. I mean, it’s, you know, I go down and get a blood draw, no big deal. (nH White male)
	Knowledge of task environment	I’m sure that it’s in my records. So, anyone that I’m seeing, you know, for care and treatment, I’m thinking they’re the ones that would be able to get my genetic testing. (Hispanic white male)	I have no problem with it. I’ve had tests done before, and it was confidential, and they kept the information between me and the VA physicians and stayed in my medical records. So no, I’m not worried about it. (African American male)	Well, I mean, with technology, the way it is, you know, your genetic DNA could get misused on other things that it’s not meant for, which I don’t know what it could be used for. But, you know, that could be something negative. It could be put in the wrong hands and be used for something negative. (African American male)
Goals	Personal Goals (distal/proximal)	I am hoping that … by getting off all these medications that I don’t really need, you know, that I can have a more clear mind, be able to, you know, function better, have a, have the ability to live a better quality of life. (AI/AN White female)	Hopefully if my medication does become less effective or develop another condition that requires medication, that I wouldn’t have to take the medication that gave me real bad side effects and could just go to the ones that work. (Hispanic White male)	Right now, I’m on three hypertensive medications, and I want to drop one. When I have my annual--it should be either this month or the end of next month--I want to talk about that again with my provider. (African American female)
Memory, attention, and decision processes	Decision-making	So, the healthcare provider suggesting it, for one, that was step one. But I guess before step one was my negative experience with side effects from medication. (nH White male)	I wasn’t exactly sure what was entailed. And, at that time, I wasn’t, didn’t feel that interested, and I didn’t do any research or background check, and so I just decided not to participate. (African American male)	I was in a negative frame of mind, and I still am regarding medicine. The prescriptions they have given me have done nothing. They have not influenced what’s going on with my [condition redacted to protect privacy] whatsoever. And so, I don’t know that genetic testing is going to help that at all. I doubt it. (nH White male)
Social influences	Social norms	She [veteran’s friend] told me about it [PGx testing] when her mental health team first suggested it. And so, we were kind of like, eh, she’s going to try this, and we’re going to kind of sit back and watch and see what happens. And her test results came back, and she sat down with her doctor and went over the test results. And they immediately switched her meds around. It was such immediate improvement, such a quick improvement after going over the results with her. (nH White Female)	N/A	N/A
Optimism	Optimism	Well, my goal is to try to stay as healthy as I can because I’m getting up there in the age where I need to stay healthy so I can stay alive. That’s my main goal: to stay healthy. I wanted to know that I was taking the right medicine so I could stay healthy. (nH White male)	There are some [benefits]. When I first started seeing a psychiatrist, I had some really bad side effects. Hopefully if my medication does become less effective or I develop another condition that requires medication, I wouldn’t have to take the medication that gave me real bad side effects and could just go to the ones that work. (Hispanic White male)	I think it’s a good opportunity, so they don’t have to try so many [medications] to get it right. (AI/AN male)
	Pessimism	N/A	N/A	In a way, I just thought it was a waste of time. I mean, it is testing me, taking blood from me. How is that going to help the next veteran? It’s just for that, the individual person, right? (nH White male)
Environmental context	Person x environment interactions Salient events/critical incidents Environmental Stressors	Well, me and my healthcare person have already been over them, and we think we’re doing real good. Nothing’s conflicting with anything, and the results came back good. So, we think we’re on the right, we’re doing the right thing. (nH White male)	It’s just, my PCP is like 25 min away, so, yeah, I mean, timing does have a little bit to do with it. And the VA hospital is about a 30-min drive away. So, if I had, you know, something 15 min away or 10 min away, it would be much easier to go over on a lunch break or something like that and get things like that taken care of. (nH White male)	I know that they can’t harm me if I don’t do it, and if I do, there could be possibilities of something coming up negatively in the future of my DNA used for this, my DNA used for that. I don’t know…The government has tested people without their knowledge and done things to the people without their knowledge. And find out later there was something negative that the government have done to people. (African American male)
Beliefs about consequences	Outcome expectancies	So, some people might view it as a waste of time or an unnecessary test, but for me it was very necessary. Because if I would have been waiting on that and took a pause and a break and waiting on those test results, I may have potentially agreed to allowing them to try some other medications and would have wound up with the same problem or worse. (Hispanic White male)	N/A	I’m just a single female. I don’t see how genetic testing is going to benefit because I’m 77 years old. I think I’ll be 78 this month. And genetic testing, I don’t understand how that’s going to work in my behalf because I am single with no children. (nH White female)
Intentions		I would do anything to help myself and to help other veterans, of course. I would do another genetic testing if it was more specific… Like specific to the medication that would work. (H White, male)	There’s a possibility. I have a lot of medical issues going on right now. So now that I have a little more information on it, if the opportunity presented itself again, then there’s a possibility that I might go ahead and do it. (African American male)	Well, I can’t predict the future, and I know that that I’m always subject to changing my mind. That’s, the older I get, the more I learn every day from my own mistakes. And it may happen that I see this as a mistake. It hasn’t yet. (nH White, male)
Emotions	Positive/negative affect, anxiety, fear	Oh, I felt that, I’m just really grateful I had that opportunity. I could cry. I mean, that’s, not everybody can get that done. I don’t know what something like that costs. I don’t have a clue, but I just feel honored that I could, and humbled that I could get that done, you know. (nH White, female)	N/A	Just kind of like just anxiety, just having that kind of, yeah, specific detail, just your genetic makeup being handed over to, you know, VA is not necessarily the most trustworthy entity. (nH White, male)
Outcomes of testing		[I feel] good, very good, because there’s not a question in the back ofmy mind if I’m going to keep trying medications, which I’m not. So that’s, for me, that’sa big thing I can check off the boxes. I’m not going to be playing around trying to figureout what concoction of chemicals works or doesn’t work. (H White, male)	N/A	N/A
Process improvement		I think it needs to be more readily available to everybody right now. (nH White female)	I think maybe if I was provided a pamphlet with information, what the testing would be used for exactly, who would have that information, know what exactly they’re looking for. (nH White male)	Maybe more information just because, and it’s like when you say we’re going to use your DNA to test, to, say, test to see if we use the right medication for the right people. It’s like, when they say it like that, it’s like a more clinical thing. Like it’s not personal. You understand? …Maybe make it a little bit more personal instead of like more cut-and-dry clinical. (African American male)

Non-Hispanic is abbreviated as nH; American Indian/Alaskan native is abbreviated as AI/AN.

## Data Availability

To request the data from the outlined paper, please contact Voils.
